# Magnetoreception in the wood mouse (*Apodemus sylvaticus*): influence of weak frequency-modulated radio frequency fields

**DOI:** 10.1038/srep09917

**Published:** 2015-04-29

**Authors:** E. Pascal Malkemper, Stephan H. K. Eder, Sabine Begall, John B. Phillips, Michael Winklhofer, Vlastimil Hart, Hynek Burda

**Affiliations:** 1 Department of General Zoology, Faculty of Biology, University of Duisburg-Essen, 45117 Essen, Germany; 2 Department of Earth and Environmental Sciences, Geophysics, Munich University, 80333 Munich, Germany; 3 Department of Biological Sciences, Virginia Tech, Blacksburg, Virginia, United States of America; 4 Faculty of Physics, University of Duisburg-Essen, 47057 Duisburg, Germany; 5 Department of Game Management and Wildlife Biology, Faculty of Forestry and Wood Sciences, Czech University of Life Sciences, 16521 Praha 6, Czech Republic; 6 Faculty of Science, University of South Bohemia, Branisovska 31, 370 05 Ceske Budejovice, Czech Republic

## Abstract

The mammalian magnetic sense is predominantly studied in species with reduced vision such as mole-rats and bats. Far less is known about surface-dwelling (epigeic) rodents with well-developed eyes. Here, we tested the wood mouse *Apodemus sylvaticus* for magnetoreception using a simple behavioural assay in which mice are allowed to build nests overnight in a visually symmetrical, circular arena. The tests were performed in the ambient magnetic field or in a field rotated by 90°. When plotted with respect to magnetic north, the nests were bimodally clustered in the northern and southern sectors, clearly indicating that the animals used magnetic cues. Additionally, mice were tested in the ambient magnetic field with a superimposed radio frequency magnetic field of the order of 100 nT. Wood mice exposed to a 0.9 to 5 MHz frequency sweep changed their preference from north-south to east-west. In contrast to birds, however, a constant frequency field tuned to the Larmor frequency (1.33 MHz) had no effect on mouse orientation. In sum, we demonstrated magnetoreception in wood mice and provide first evidence for a radical-pair mechanism in a mammal.

The ability to sense the geomagnetic field for use as a global reference during spatial orientation has been demonstrated in more than 30 species of vertebrates^1^. The majority of studies have been performed on birds and have yielded valuable insights into the underlying magnetoreception mechanism^2^. Extensive research has also been carried out on the detection and use of magnetic cues in amphibians and turtles e.g.^3^,^4^,^5^. In mammals, research on magnetoreception is far less advanced, but the existing data support the idea of a phylogenetically ancient sense^6^,^7^, with magnetosensitivity being reported in an increasing number of species including representatives of at least five different orders (reviewed in^8^).

Early homing and orientation experiments with epigeic rodents suggested that they might possess a magnetic compass used for navigation^9^,^10^. Later studies, however, challenged these findings with negative results and failed experimental replications^11^,^12^. It was not until the early 1990s that a robust behavioural assay was developed that provided solid and replicable evidence for magnetoreception in mammals: the nest building assay^13^. This paradigm, in which the directions (positions) of nests built along the wall of a circular (radially symmetrical) arena relative to the magnetic field are analysed, has been used ever since with a variety of species, and has yielded the first insights into the mechanisms of magnetoreception in mammals^14^,^15^,^16^,^17^,^18^,^19^,^20^,^21^,^22^.

The available evidence indicates that there are two biophysically distinct magnetoreception mechanisms in terrestrial vertebrates: magnetic particles and light-dependent biochemical reactions that involve radical pair intermediates. The magnetic particle mechanism assumes that the torque or force exerted by the Earth’s magnetic field on particles of magnetite (or its oxidized form: maghemite) is transduced through direct effects on membrane conductivity^23^ or through the opening of membrane channels mediated by filamentous connections or via membrane deformation^24^,^25^,^26^. The radical pair mechanism of magnetoreception is based on an effect of an earth-strength magnetic field on the singlet-triplet interconversion rate of a spin-correlated radical pair formed after photo-excitation (reviewed in^27^,^28^). A specialized class of retinal photopigments, cryptochromes, the only animal photopigments known to form radical pair intermediates, are thought to be involved in the radical pair mechanism, leading to the suggestion that the magnetic field may be perceived as a visual pattern superimposed on the animal’s surroundings^29^. As this pattern would be axially symmetric, it is consistent with the inclination compass found in birds and amphibians that uses the slope direction of the field lines to distinguish polewards and equatorwards, rather than northwards and southwards as in a true polarity compass^4^,^30^. Studies indicate, however, that the two mechanisms, magnetic particle mechanism and radical pair mechanism, are not mutually exclusive, but at least birds and amphibians might have both mechanisms that they use in different behavioural contexts and/or to provide spatial and directional information^4^,^31^.

For rodents (and mammals in general) the majority of behavioural and histological findings published so far have only provided evidence for the involvement of a magnetic particle mechanism. The compass of Ansell’s mole-rats is polarity sensitive (i.e., able to distinguish magnetic north from south)^21^. Also, the magnetic compass of mole-rats is affected by brief magnetic pulses with an intensity high enough to re-magnetize magnetite particles^32^. The same properties were found for the magnetoreceptors of microphthalmic bats^33^. Importantly, the magnetic compass of Ansell’s mole-rats was unaffected by a treatment specifically designed to perturb the radical pair mechanism using a RF field in the low MHz range (broadband as well as Larmor frequency, i.e., 1.315 MHz)^16^ over 300 times stronger than that shown to cause disorientation in birds^34^,^35^,^36^,^37^,^38^,^39^.

More recent studies, however, raised the question of whether epigeic rodents (i.e., species that unlike mole-rats are mostly active above ground) could have a radical pair mechanism-based magnetic compass. A nest building study of C57BL/6 mice has provided evidence that these mice rely on a magnetic compass that exhibits an axially symmetrical pattern of response, consistent with a radical pair mechanism of magnetoreception^18^. In a more recent study, C57BL/6 laboratory mice were trained to remember the magnetic compass direction of a submerged platform in a variation of the classic Morris water maze assay, but the training was only successful in the water maze study after the test room had been electromagnetically shielded against ambient (i.e., man-made) radio frequency (RF) noise in the frequency range between 0.1 and 100 MHz with residual values lower than 0.1 nT^40^. Electromagnetic shielding was also reported to be necessary for the stable compass responses in the earlier nest building studies of C57BL/6J mice^18^ and Siberian hamsters^22^. Therefore, the current state of knowledge does not allow firm conclusions to be drawn about the involvement of a radical pair mechanism in magnetoreception in epigeic rodents, or in other mammalian orders apart from mole-rats.

In this study we addressed two questions. First, do wood mice, *Apodemus sylvaticus*, have a magnetic sense, as suggested by early orientation experiments^10^? Second, if they have a magnetic sense, is it sensitive to weak RF magnetic fields, which would point to the involvement of a radical pair mechanism? To answer these questions, we tested freshly captured animals with the classic nest building assay and exposed them to different alignments of an earth-strength magnetic field and to different low intensity RF-fields.

## Results

### Nest building preference in *Apodemus sylvaticus*

**Figure 1:**
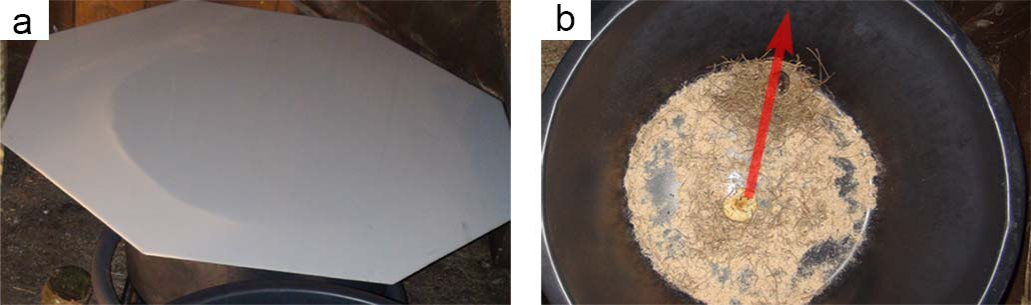
The nest building assay. a. The arena (50 cm diameter) was prepared with hay and saw dust as nest building material and an apple slice and grain as food. A wood mouse was placed in the arena and the setup was covered with a frosted white PVC-sheet overnight. b. On the next day the direction of the nest was measured as from the centre of the arena (arrow).

**Figure 2:**
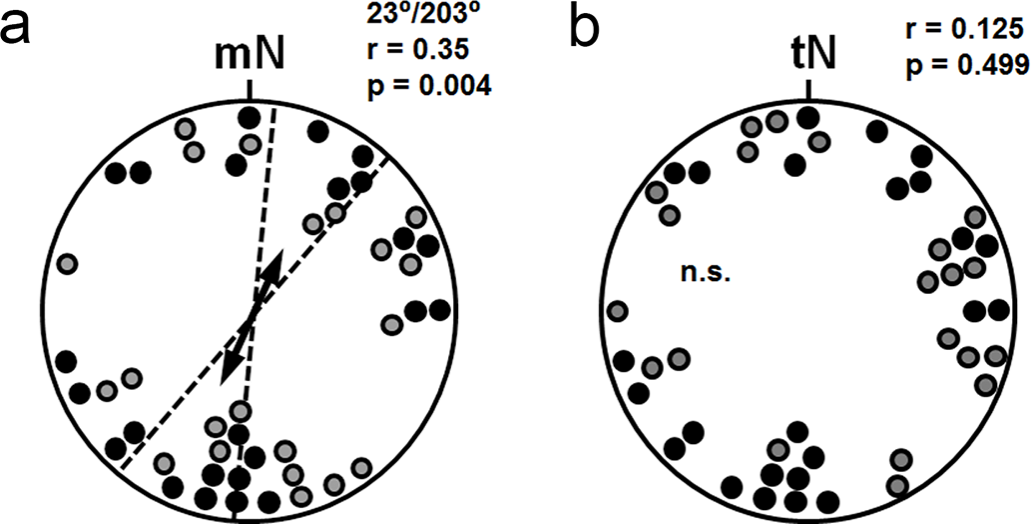
Orientation of wood mice nests built in a visually symmetrical circular arena. Each data point in the circular diagrams (upper panel) represents the position of a nest built by an individual mouse. The figure shows the pooled nest positions of mice tested in the ambient field (filled circles) and with magnetic north shifted 90° counterclockwise (open circles). a. Bearings relative to magnetic north (mN) in the arena. b. Absolute bearings in the arena. Arrows give the mean vector for the distribution of the nests, the dotted lines are the 95% confidence intervals for the mean bearing (µ) of non-random distributions (p-value of the Rayleigh test is given for each distribution). The double-headed arrows indicate bimodal distributions; the lengths of the arrows represent the mean vector length r (scaled so the radius of the circles corresponds to r = 1), which provides a measure of the degree of clustering in the distribution of the bearings. n.s. = not significant

The vast majority of tested mice built a single and clearly distinguishable nest at the wall of the arena overnight (Figure 1). Plotting the nest directions of nests built under control conditions (no RF) in the ambient (intensity: 49.03 µT) and the 90° rotated magnetic field (intensity: 49.20 µT) with respect to magnetic north revealed highly significant bimodal orientation along the north-northeast and south-southwest magnetic axis (Figure 2a). In contrast, plotting the nests with respect to topographic north (i.e. the absolute directions of the nests ignoring the alignment of the magnetic field) failed to reveal a significant clustering of bearings (Figure 2b). The distributions of nests built in the two magnetic conditions were significantly different (Table 1; Watson U^2^: p < 0.01, U^2^ = 0.293).

### Influence of radio frequency magnetic fields

**Figure 3:**
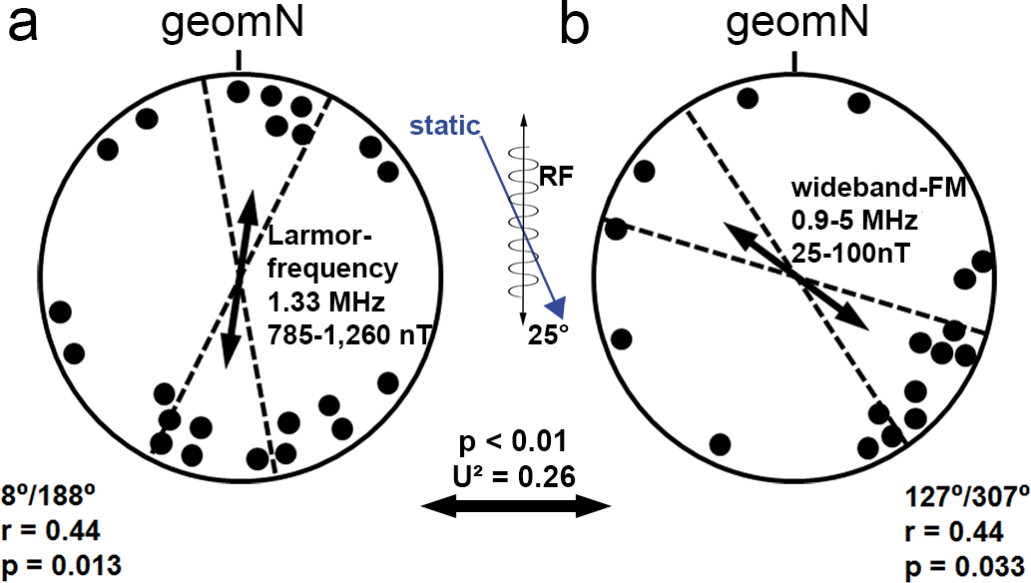
Orientation of wood mice nests built in a visually symmetrical circular arena in the ambient geomagnetic field with superimposed RF magnetic fields. The oscillating fields were aligned vertically at an angle of 25° to the geomagnetic field lines. Each data point represents the position of a nest built by an individual mouse. a. Bearings relative to geomagnetic north (geomN) in the arena in the ambient magnetic field with a superimposed Larmor frequency oscillating field (785 – 1,260 nT). b. Bearings relative to geomagnetic north (geomN) in the arena in the ambient magnetic field with a superimposed wideband (0.9–5 MHz) oscillating field (25–100 nT). Arrows give the mean vector for the distribution of the nests, the dotted lines are the 95% confidence intervals for the mean bearing (µ) of non-random distributions (p-value of the Rayleigh test is given for each distribution). The double-headed arrows indicate bimodal distributions; the lengths of the arrows represent the mean vector length r (scaled so the radius of the circles corresponds to r = 1), which provides a measure of the degree of clustering in the distribution of the bearings. The apparent unimodal preference for the southeast in the wideband-FM condition (b) was not significant (angular analysis: µ = 130° ± 78°, r = 0.398; Rayleigh test: p = 0.066, Z = 2.696). Significant differences between the distributions are indicated by the p- and U^2^-values of the Watson U^2^ test. n.s. = not significant

In both RF conditions the oscillating fields were aligned vertically at an angle of 25° to the geomagnetic field lines. Nests built in the ambient magnetic field (intensity: 49.05 µT) under the influence of a Larmor frequency oscillating magnetic field (1.33 MHz, 785 – 1,260 nT) showed a significant bimodal distribution of bearings clustered along the north-south axis (Figure 3a) that was indistinguishable from controls (Watson U^2^: p > 0.5, U^2^ = 0.05).

In contrast, animals tested in a wideband-FM field (frequency sweep from 0.9–5 MHz, 25–100 nT, at one msec intervals) displayed a dramatic change in the distribution of nests relative to the geomagnetic field, with the axis of orientation rotated by approximately 90°. The mice now built their nests in the northwest and southeast sectors with a clear preference for the latter (Figure 3b). Importantly, the nests built under the two RF conditions showed significantly different distributions (Watson U^2^: p < 0.01, U^2^ = 0.26).

## Discussion

The results provide clear evidence for a magnetic sense in wood mice. When building a nest in an otherwise featureless environment, the animals exhibited a spontaneous preference for the magnetic north-northeast and south-southwest axis. These findings are consistent with the earlier report of a magnetic compass sense in this species by Mather and Baker^10^. A magnetic compass would be highly beneficial for nocturnal wood mice that occupy comparatively large home ranges of 1–2 ha^41^, perform regular foraging bouts over distances of more than 200 m, and show remarkable homing ability from unfamiliar locations after displacements of up to 350 m^42^,^43^.

It is unclear whether the observed preference is part of a homing response or a spontaneous directional preference. However, even though many animals used in this study were caught north of the testing site, none were caught south of it. This renders it unlikely that the observed axial preference is a homing response. However, the observed directional preference is consistent with the spontaneous bimodal magnetic alignment observed in other vertebrates (reviewed in^44^). Concordantly, laboratory mice, in addition to showing learned compass orientation relative to the magnetic field, also exhibit a weak, presumably innate (i.e., independent of any learned direction) axial preference along the magnetic north-south axis^18^. Recently, such a preference was also revealed in the semi-fossorial bank vole (*Myodes glareolus*), which the authors suggested is likely to be innate^15^. Consequently, although there is insufficient information to determine if the observed preference in wood mice is innate or learned, it appears likely that at least some component of the response is innate. Although the adaptive significance of spontaneous magnetic alignment remains an open question^8^,^44^,^45^,^46^, the widespread occurrence in epigeic mammals makes this response ideal for initial studies of the mechanism(s) of magnetoreception.

**Figure 4:**
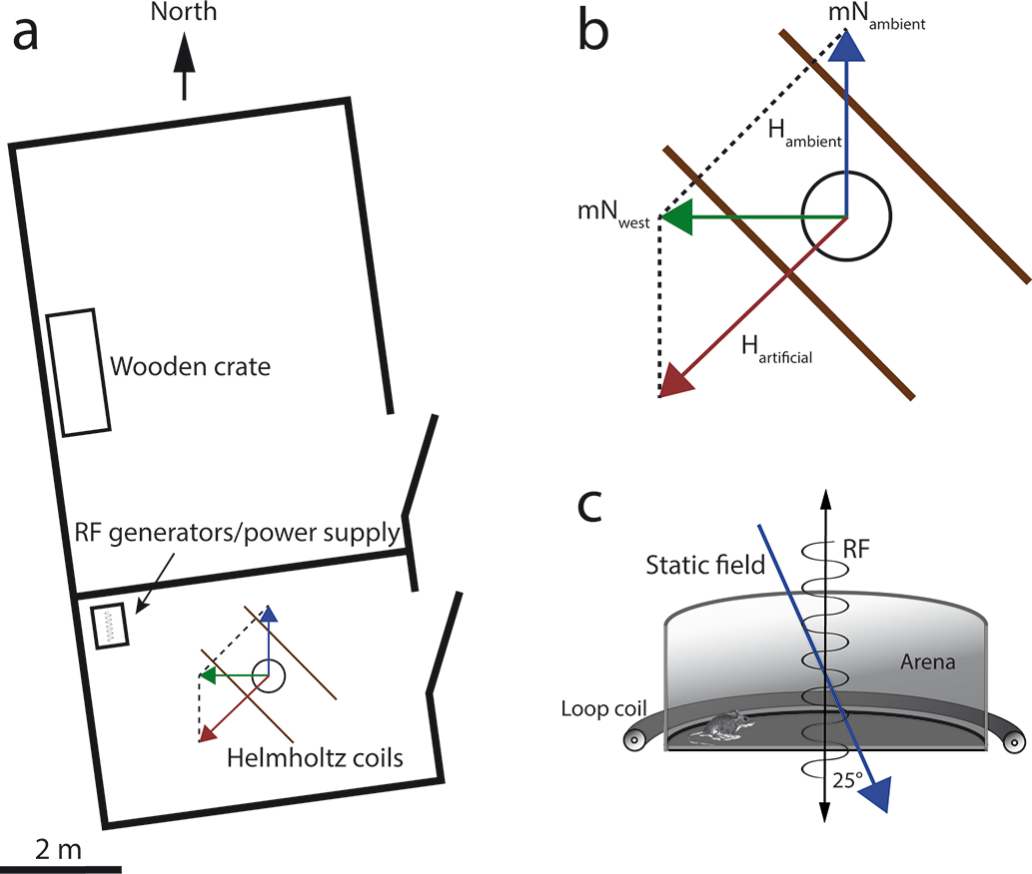
Overview of the testing site, the coil-setup and the nest-building arena. a. Top view of the empty horse stable that consisted of two compartments. One compartment contained the wooden enclosure, the other one the testing setup and coil systems. b. Production of the artificial static magnetic field (top view). In the control condition (mN_ambient_) the horizontal of the ambient magnetic field (H_ambient_) was left unchanged. To shift magnetic north by 90° counterclockwise (mN_west_), an artificial magnetic field was added with a 135° clockwise aligned Helmholtz-coil pair (H_artificial_) to produce a 90° shifted resultant field of the same inclination and total intensity as the ambient magnetic field. c. Profile of the test arena and the loop coil used to produce the oscillating magnetic fields in the RF range. The oscillating fields were aligned vertically at an angle of 25° to the static field lines. The scale only applies to part a. of the figure.

The available evidence indicates that subterranean, microphthalmic mole-rats rely on a light-independent and RF-insensitive magnetic particle based mechanism of magnetoreception^16^. On the other hand, the properties of learned magnetic compass orientation by epigeic rodents are consistent with the involvement of a radical pair mechanism^40^. This suggests that the visual ecology/physiology (adaptation to light levels available to diurnal and nocturnal animals active above ground, rather than to the absence of light in the subterranean ecotope), rather than phylogenetic relatedness (membership in the class Mammalia), may be the principle set of factors influencing the type of magnetoreception mechanism used to obtain directional (i.e., compass) information. To determine if macrophthalmic, epigeic wood mice indeed have a radical pair mechanism, we tested the sensitivity of wood mice to low level radio frequency fields, using the types of stimuli used in earlier studies of birds and mole-rats^16^,^35^,^47^, i.e., both the Larmor frequency and wideband-FM (comparable to broadband-RF used in other studies) oscillating magnetic fields. Contrary to earlier studies of migratory birds^35^,^47^, wood mice exhibited non-random directional preferences in both conditions: The distribution of bearings obtained from mice tested under the Larmor frequency condition was indistinguishable from controls (i.e., nests were bimodally distributed approximately along the north-south magnetic axis; Figure 3a), while that of mice exposed to the wideband frequency sweep was rotated by roughly 90° (Figure 3b). As the angle between the static field and the RF fields was the same in both RF conditions (see Figure 4c), the only differences were the overall intensity, the temporal pattern, and the frequency spectrum. The intensities in the Larmor frequency condition had minimum values of 785 nT in the centre of the arena and therefore greatly exceeded those shown to affect the inclination compass of birds^39^,^47^. In the wideband-FM condition the RF intensities were lower, due to the frequency response characteristics of the coil. They varied across the frequency range between 25–50 nT in the centre of the coil to twice these values at the periphery of the arena. These field strengths are comparable to the ones used in Engels et al.^39^, who were able to disrupt the magnetic compass orientation of European robins by broadband electromagnetic noise with a spectral intensity of 0.1–0.2 nT per 10 kHz in the range of 600 kHz–3 MHz, which upon integration over the frequency domain translates into a RF magnetic field amplitude of 30–35 nT in the time domain.

The results are in accordance with a radical pair mechanism of magnetoreception, providing for the first time positive evidence for such a mechanism in a mammal. Even though we cannot rule out completely that the mice were affected by both RF treatments and oriented topographically in the Larmor frequency field, the similarity between nest distribution in the latter and the orientation in the unchanged geomagnetic field suggests that the wood mice magnetic receptors were unaffected. Future experiments will hopefully verify this. For now, the fact that we observed an effect on nest positioning under a low intensity wideband-FM field, but no (behavioural) effect under the Larmor frequency field at effectively a 15–30 times higher intensity leads us to speculate about the underlying mechanism. The response is consistent with a radical pair mechanism in which both electron spins have an anisotropic coupling to their respective host molecules, due either to nuclear hyperfine interactions^29^,^48^,^49^ or, less likely, to spin-orbit coupling^50^. If we make the assumption that cryptochrome 1^51^,^52^ is the molecule responsible for the radical pair mechanism also in mammals, the radical partner of the flavin adenine dinucleotide (FAD) cofactor thus could not be a reactive oxygen species (superoxide), as has been suggested for birds^28^,^53^, because superoxide is free of hyperfine interactions. The findings are rather compatible with the radical partner being tryptophan as in the original radical pair mechanism model^29^ or ascorbyl as recently proposed by Lee et al.^54^. Of course, the results do not exclude that the host molecule in mammals and perhaps other taxa might differ from that in birds. In either case, a large number of resonance frequencies, both below and above the Larmor frequency, are only possible in a radical pair mechanism in which both members of the radical pair have hyperfine interactions, which would then have been excited by the frequencies in the wideband-FM condition.

Interestingly, while RF magnetic fields so far have been found to cause disorientation in birds, in the present experiments the wideband-FM field caused re-orientation in mice, with nest-building positions shifted by approximately 90° relative to the axis of orientation observed in the ambient magnetic field. There are two possible explanations for this re-orientation. First, the wideband-RF might have disrupted input from the radical pair mechanism, causing the mice to rely on an alternative source of directional information (e.g., non-magnetic, or magnetic particle mechanism-based comparable to the fixed direction response of birds tested in unnatural light conditions^37^,^55^). Alternatively, the wideband-RF may have altered, rather than eliminated, the pattern of radical pair mechanism response, as it has recently been proposed as an explanation for an effect of a Larmor frequency magnetic field on spontaneous magnetic alignment in turtles^56^. To our knowledge, the possibility of this type of RF effect has not yet been addressed by the available models of the radical pair mechanism. For simple reference-probe radical pair mechanism models it is the lifetime of the spin-correlated radical pair (“spin correlation time”) that determines the magnitude of the effect of a weak RF magnetic field on the radical pair dynamics: long correlation times, in the order of 100 microseconds allow weak RF magnetic fields to fully perturb the singlet-triplet interconversion, leading to a flattened angular response (suppression of the directional dependence required for a compass). Shorter correlation times alter the absolute values of the yield without flattening the angular response^50^,^57^. Theoretically, it is possible that the radical pair mechanism in rodents is based on a radical pair with shorter spin coherence time than the ones in migratory birds, so that the effect of a RF magnetic field could be different in the two taxa. The altered response in the short-lived radical pair would be equivalent to the response produced by an intensity shift in the static magnetic field^57^, which could produce a change in the magnetic visual pattern.

It has been proposed that vertebrates might exploit the visual pattern of response produced by the radical pair mechanism as a global reference that could function as a simple spherical grid or coordinate system fixed in alignment relative to the magnetic field that appears as a visual pattern superimposed on the animal’s surroundings^58^. Such a reference system would be useful in a variety of daily challenges from integrating spatial information from multiple sensory modalities, in novel surroundings, to improving 3-dimensional path stabilization^59^ and course control^60^, to placing multiple locales into register to form a global map of familiar space^46^,^58^. If the magnetic field is perceived in this way in epigeic rodents, mice might position themselves and/or their nests in a specific alignment with respect to the pattern generated by the radical pair mechanism. Consequently, a change in the ‘visual’ pattern caused by RF treatment could result in a corresponding change in the distribution of nest positions.

It is widely believed that RF magnetic fields influence exclusively a radical pair mechanism, not a magnetic particle mechanism. This is certainly true for single-domain magnetite, where the inertia of the particles surrounded by the viscous cytoplasm is generally believed to hinder motion and thus transduction of oscillating fields in the radio frequency range^36^,^61^. However, according to Shcherbakov & Winklhofer^25^, a magnetic particle mechanism based on magnetic susceptibility, such as the maghemite-superparamagnetic magnetite hybrid magnetoreceptor proposed by Fleissner et al.^62^ would convert the radiation into thermal agitation. As with the putative effect on a radical pair mechanism, it is not clear why such a heating effect would cause re-orientation, rather than disorientation. Importantly, however, due to the higher intensity of the Larmor frequency stimulus compared to the wideband stimulus, any heating effect would have been more pronounced for the Larmor frequency condition. Consequently, the finding of an effect of the wideband RF stimulus, but not of the higher intensity Larmor frequency stimulus, argues against a nonspecific (i.e., thermal) effect on a mechanism or process other than the radical pair mechanism.

In sum, we show that wood mice possess a magnetic sense that they use to position their nests along the NNE-SSW axis relative to the magnetic field. The NNE-SSW preference was not altered by RF fields delivered at the Larmor frequency, but was shifted by approximately 90° by a RF frequency sweep (0.9–5 MHz repeated at 1 kHz) at an intensity of only ~5% that of the Larmor frequency stimulus. The results point to the involvement of a radical pair mechanism, the first such evidence for a mammal, although further research is needed to provide a more thorough characterization of the underlying mechanism. Finally and importantly, it should be noted, that the RF magnetic fields applied here have peak intensities below the ICNIRP guidelines for general public exposure (63, i.e., *B*_rms_ = 0.92 µT/f [MHz], or *B*_peak_ = 1.30 µT/f [MHz]) considered as harmless for human health. Yet, we show that they are sufficient to affect behaviour in a mammal.

## Methods

### Static magnetic fields

We used a pair of double-wrapped Helmholtz-coils (2.15 m diameter, 10+10 turns of 14 gauge copper wire wrapped on a wooden frame, current 1.76 A) powered by a current-regulated power supply (Manson DPD-3030) to alter the direction of the geomagnetic field. Parallel current flow through both wires on the coils created a magnetic field, while with the current flowing antiparallel no magnetic field was created but possible side effects (heat, vibrations, electric fields) where the same in both conditions^64^. The coils were arranged in such a way that magnetic north could be shifted by 90° counterclockwise without changing the intensity or inclination of the geomagnetic field (Figure 4a,b;^3^). The intensity of the static field and of extremely low-frequency (mainly 50 Hz) oscillating fields was measured with a 3-axial magnetometer (Gigahertz NFA 1000) equipped with an additional magnetostatic probe (Gigahertz MS-NFA). The intensity of the local magnetic field within the arena was 49.05 µT. With antiparallel current flow the overall intensity was 49.03 µT and it was 49.20 µT when north was shifted westwards. The output of the power supply was run through an EMI filter (Schurter 5500.2058) before entering the coils to reduce low RF fields. Residual oscillating magnetic fields were detectable in both static field conditions and mainly in the 150–180 Hz range, with intensities around 60 nT (parallel current flow) and 6 nT (antiparallel current flow).

### Radio frequency magnetic fields

Split-shield magnetic-field loops for RF experiments were constructed using coaxial cable (Aircell 7). One loop was designed with a sharp resonance at about 1.3 MHz for application of a magnetic field oscillating at the local Larmor frequency (1.33 MHz) with a 47 Ohm resistance at the feed for impedance matching, the other cable had a broad resonance between 0.9–5 MHz, with maximum at 4 MHz, where the magnetic field was two times stronger than at 0.9 MHz. The coils (60 cm diameter) were powered using a Wavetek 144 function generator for a 1.33 MHz continuous sine wave and a Wavetek 193 sweep generator used for generating a wideband-frequency modulated (FM) field, where the frequency sweep (0.9 MHz to 5 MHz) was repeated at intervals of 1 msec. The oscillating fields were aligned vertically at an angle of 25° to the static field lines (Figure 4c). The RF field produced by the coils was measured with an ETS-Lindgren split-shield magnetic-field probe (7405 E&H 6 cm diameter near field loop probe) connected through a coax cable to an oscilloscope (Picoscope 4224). The intensities in the Larmor frequency condition had maximum values of 1.26 µT in the periphery of the arena and minimum values of 785 nT in the centre, while in the wideband-FM condition, due to the frequency response characteristics of the coil, the intensities varied between 25 nT-50 nT in the centre of the coil to twice these values at the periphery of the arena. An induction coil connected to a HAMEG (HMO 3524) oscilloscope was used (in FFT mode) to monitor the low-frequency magnetic noise. Neither single-frequency nor the sweeping conditions were found to cause an enhanced noise level in the low-frequency range.

### Experimental procedure

The experiments were performed in an empty horse stable in a rural area of the Bohemian Forest, Czech Republic (49°9′10.28"N, 13°20′56.45"E) in summer and autumn of 2013. Recently, Engels et al.^39^ showed that anthropogenic electromagnetic noise of spectrum level intensities a low as 1 nT disturbed the magnetic compass of European robins when being tested in unshielded wooden huts at the campus of the University of Oldenburg. Although we tested the wood mice in an unshielded environment, the testing site was remote and comparable to the rural locality used by Engels and colleagues where the European robins were well oriented.

Wood mice (*Apodemus sylvaticus*) were live-trapped on private property within the vicinity of the stable by means of see-saw traps. The trapping and all experiments were approved by the Institutional Animal Care and Use Committee of the University of South Bohemia, and the Ministry of Education, Youth and Sports of the Czech Republic (no. 7946/2010-30) and all experiments were performed in accordance with their guidelines and regulations. Until testing, the wood mice were kept in a rectangular wooden enclosure (approx. 2 m × 1 m × 1 m) based in an adjacent part of the stable (Figure 4a) and fed apples and grain and given access to water *ad libitum*. For habituation and stress reduction, each mouse was kept for at least one night but no longer than three nights in the enclosure before being tested.

The animals were tested in a circular arena (diameter 50 cm) made of black PVC. The floor of the arena was evenly covered with sawdust and hay which served as nest building material. An apple slice (1–2 cm thick) and some grain placed in the centre of the arena served as food and water supply (Figure 1a). All experiments started in the evening and were conducted overnight. The magnetic conditions (control, 90° shift, RF) had been set before the mouse was introduced into the arena. The condition for each day was randomly chosen. The mice were released into the middle of the arena and the arena was quickly covered with a translucent frosted white PVC-sheet (Figure 1b). The next morning, the direction of the nest was measured with a hand-held compass (Figure 1c) and the mouse was released. Only nests built against the wall of the arena (max. distance 10 cm) were counted. Nights with thunderstorms were excluded from the analysis (confer^40^). After each test, the arena was emptied and thoroughly cleaned with 70% ethanol.

To control for a possible observer bias, a subset of the nests (n = 24) was analysed in a double-blind fashion: pictures of the nests were taken with a digital camera from above and later analysed by a second person unaware of magnetic north and the experimental conditions. The mean difference between the nest directions obtained from direct compass measurements and those taken from the pictures was 3.7° (circular SD = 7.4°).

### Statistical analysis

We used standard circular statistics to analyse the distributions of the nest positions^65^. All calculations were carried out with Oriana 4.02 (Kovach Computing). Each nest direction was treated as an independent data point. The likelihood of retesting a mouse was low because wood mice avoid traps for some time after they have been captured (unpublished observations). Furthermore, any mouse that was recaptured had only a 25% chance of being tested in the same experimental condition. Mean vectors were calculated by vector addition. The method of doubling the angles was used to convert angular data in axial data prior to statistical analysis. The Rayleigh test was employed to test the data for significant deviation from random distribution with p<0.05 as the threshold of statistical significance. The distributions of the nests in different conditions (group comparisons) were compared with the Watson U^2^ test employed on doubled angles.

## Author Contributions

Design of the study: EPM, SHKE, MW. Data collection: EPM, VH. Data analysis: EPM, JBP. Writing the paper: EPM, SHKE, SB, MW, JBP, HB. All authors read and approved the final version of the manuscript. We thank three reviewers for comments on an earlier version of this manuscript.

## Supplementary Material

Supplementary Information Supplementary Table S1

## Figures and Tables

**Table 1:** Directions of wood mice nests built in different magnetic conditions. Statistically significant differences between the distributions are indicated in the last column. FM = frequency-modulated, n = number of nests, CCW = counterclockwise

▒					

## References

[b1] Wiltschko R. & Wiltschko W. in *Sensing in Nature*. (ed López-Larrea C., ed. ) 126–141 (Springer, 2012).

[b2] Wiltschko R. & Wiltschko W. Avian magnetic compass: Its functional properties and physical basis. Current Zoology 56, 265–276 (2010).

[b3] Phillips J. B. Magnetic compass orientation in the eastern red-spotted newt (*Notophthalmus viridescens*). J. Comp. Physiol. A. 158, 103–109 (1986). 3723427 10.1007/BF00614524

[b4] Phillips J. B. Two magnetoreception pathways in a migratory salamander. Science 233, 765–767 (1986). 3738508 10.1126/science.3738508

[b5] Lohmann K. J., Lohman C. M. F., Ehrhart L. M., Bagley D. A. & Swing T. Geomagnetic map used in sea–turtle navigation. Nature 428, 909–910 (2004). 15118716 10.1038/428909a

[b6] Kirschvink J. L., Walker M. M. & Diebel C. E. Magnetite-based magnetoreception. Curr. Opin. Neurobiol. 11, 462–467 (2001). 11502393 10.1016/s0959-4388(00)00235-x

[b7] Kobayashi A. & Kirschvink J. L. in *Electromagnetic Fields: Biological Interactions and Mechanisms*. (ed Blank M., ed. ) 367–394 (American Chemical Society Books, 1995).

[b8] Begall S., Burda H. & Malkemper E. P. in *Adv*. Study Behav. Vol. 46 (ed Naguib M., ed. ) 45–88 (Elsevier, 2014).

[b9] August P. V., Ayvazian S. G. & Anderson J. G. T. Magnetic orientation in a small mammal, *Peromyscus leucopus*. J. Mammal. 70, 1–9 (1989).

[b10] Mather J. G. & Baker R. R. Magnetic sense of direction in woodmice for route-based navigation. Nature 291, 152–155 (1981).

[b11] Madden R. C. & Phillips J. B. An attempt to demonstrate magnetic compass orientation in two species of mammals. Learn. Behav. 15, 130–134 (1987).

[b12] Sauvé J. P. Analyse de l’orientation initiale dans une expérience de retour au gîte chez le mulot, *Apodemus sylvaticus*. Sciences et Techniques de l’Animal de Laboratoire 13, 89–91 (1988).

[b13] Burda H., Marhold S., Westenberger T., Wiltschko R. & Wiltschko W. Evidence for magnetic compass orientation in the subterranean rodent *Cryptomys hottentotus* (Bathyergidae). Experientia 46, 528–530 (1990). 2347407 10.1007/BF01954256

[b14] Oliveriusová L., Němec P., Králová Z. & Sedláček F. Magnetic compass orientation in two strictly subterranean rodents: learned or species-specific innate directional preference? J. Exp. Biol. 215, 3649–3654 (2012). 22855619 10.1242/jeb.069625

[b15] Oliveriusová L., Němec P., Pavelková Z. & Sedláček F. Spontaneous expression of magnetic compass orientation in an epigeic rodent: the bank vole, *Clethrionomys glareolus*. Naturwissenschaften 101**,** 557–563 (2014). 24913128 10.1007/s00114-014-1192-0

[b16] Thalau P., Ritz T., Burda H., Wegner R. E. & Wiltschko R. The magnetic compass mechanisms of birds and rodents are based on different physical principles. J. R. Soc. Interface 3, 583–587 (2006). 16849254 10.1098/rsif.2006.0130 PMC1664646

[b17] Wegner R. E., Begall S. & Burda H. Magnetic compass in the cornea: local anaesthesia impairs orientation in a mammal. J. Exp. Biol. 209, 4747–4750 (2006). 17114407 10.1242/jeb.02573

[b18] Muheim R., Edgar N. M., Sloan K. A. & Phillips J. B. Magnetic compass orientation in C57BL/6J mice. Learn. Behav. 34, 366–373 (2006). 17330527 10.3758/bf03193201

[b19] Marhold S., Beiles A., Burda H. & Nevo E. Spontaneous directional preference in a subterranean rodent, the blind mole-rat, *Spalax ehrenbergi*. Folia Zool. 49, 7–18 (2000).

[b20] Burda H. *et al.* Magnetic orientation in subterranean mole rats of the superspecies *Spalax ehrenbergi*: Experiments, patterns, and memory. Isr. J. Zool. 37, 182–183 (1991).

[b21] Marhold S., Wiltschko W. & Burda H. A magnetic polarity compass for direction finding in a subterranean mammal. Naturwissenschaften 84, 421–423 (1997).

[b22] Deutschlander M. E. *et al.* Learned magnetic compass orientation by the Siberian hamster, *Phodopus sungorus*. Anim. Behav. 65, 779–786 (2003).

[b23] Kirschvink J. L. & Gould J. L. Biogenic magnetite as a basis for magnetic field detection in animals. Biosystems 13, 181–201 (1981). 7213948 10.1016/0303-2647(81)90060-5

[b24] Davila A. F., Fleissner G., Winklhofer M. & Petersen N. A new model for a magnetoreceptor in homing pigeons based on interacting clusters of superparamagnetic magnetite. Phys. Chem. Earth 28, 647–652 (2003).

[b25] Shcherbakov V. P. & Winklhofer M. Theoretical analysis of flux amplification by soft magnetic material in a putative biological magnetic-field receptor. Phys. Rev. E. 81, 031921 (2010). 10.1103/PhysRevE.81.031921 20365784

[b26] Winklhofer M. & Kirschvink J. L. A quantitative assessment of torque-transducer models for magnetoreception. J. R. Soc. Interface 7, 273–289, 10.1098/rsif.2009.0435.focus (2010). PMC2843997 20086054

[b27] Dodson C. A., Hore P. & Wallace M. I. A radical sense of direction: signalling and mechanism in cryptochrome magnetoreception. Trends Biochem. Sci. 38, 435–446, http://dx.doi.org/10.1016/j.tibs.2013.07.002 (2013). 23938034 10.1016/j.tibs.2013.07.002

[b28] Ritz T., Ahmad M., Mouritsen H., Wiltschko R. & Wiltschko W. Photoreceptor-based magnetoreception: optimal design of receptor molecules, cells, and neuronal processing. J. R. Soc. Interface 7, 135–146 (2010). 10.1098/rsif.2009.0456.focus PMC2843994 20129953

[b29] Ritz T., Adem S. & Schulten K. A model for photoreceptor-based magnetoreception in birds. Biophys. J. 78, 707–718 (2000). 10653784 10.1016/S0006-3495(00)76629-X PMC1300674

[b30] Wiltschko W. & Wiltschko R. The magnetic compass of European robins. Science 176, 62–64 (1972). 17784420 10.1126/science.176.4030.62

[b31] Wiltschko R. & Wiltschko W. The magnetite-based receptors in the beak of birds and their role in avian navigation. J. Comp. Physiol. A 199, 89–98 (2012). 10.1007/s00359-012-0769-3 PMC3552369 23111859

[b32] Marhold S., Burda H., Kreilos I. & Wiltschko W. in *RIN 97 Orientation & Navigation - Birds, Humans and Other Animals*. 1–9 (Royal Institute of Navigation London).

[b33] Holland R. A., Kirschvink J. L., Doak T. G. & Wikelski M. Bats use magnetite to detect the Earth's magnetic field. PLoS. ONE 3, e1676 (2008). 18301753 10.1371/journal.pone.0001676 PMC2246016

[b34] Keary N. *et al.* Oscillating magnetic field disrupts magnetic orientation in Zebra finches, *Taeniopygia guttata*. Front. Zool. 6, 25 (2009). 19852792 10.1186/1742-9994-6-25 PMC2774300

[b35] Ritz T., Thalau P., Phillips J. B., Wiltschko R. & Wiltschko W. Resonance effects indicate a radical-pair mechanism for avian magnetic compass. Nature 429, 177–180 (2004). 15141211 10.1038/nature02534

[b36] Rodgers C. T. & Hore P. J. Chemical magnetoreception in birds: the radical pair mechanism. Proc. Natl. Acad. Sci. U.S.A. 106, 353–360 (2009). 19129499 10.1073/pnas.0711968106 PMC2626707

[b37] Stapput K., Thalau P., Wiltschko R. & Wiltschko W. Orientation of birds in total darkness. Curr. Biol. 18, 602–606 (2008). 18424144 10.1016/j.cub.2008.03.046

[b38] Thalau P., Ritz T., Stapput K., Wiltschko R. & Wiltschko W. Magnetic compass orientation of migratory birds in the presence of a 1.315 MHz oscillating field. Naturwissenschaften 92, 86–90 (2005). 15614508 10.1007/s00114-004-0595-8

[b39] Engels S. *et al.* Anthropogenic electromagnetic noise disrupts magnetic compass orientation in a migratory bird. Nature 509, 353–356, 10.1038/nature13290 (2014). 24805233

[b40] Phillips J. B. *et al.* Rapid learning of magnetic compass direction by C57BL/6 mice in a 4-armed ‘plus’ water maze. PLoS. ONE 8, e73112, 10.1371/journal.pone.0073112 (2013). 24023673 PMC3758273

[b41] Tew T. E. & MacDonald D. W. Dynamics of space use and male vigour amongst wood mice, *Apodemus sylvaticus*, in the cereal ecosystem. Behav. Ecol. Sociobiol. 34, 337–345 (1994).

[b42] Hacker H. P. & Pearson H. S. Distribution of the long-tailed field mouse, *Apodemus sylvaticus*, on South Haven Peninsula, Dorset, in 1937, with some observations on its wandering and homing powers. J. Proc. Linn. Soc., Zool. 42, 1–17, 10.1111/j.1096-3642.1951.tb01850.x (1951).

[b43] Jamon M. & Bovet P. Possible use of environmental gradients in orientation by homing wood mice, *Apodemus sylvaticus*. Behav. Process. 15, 93–107, http://dx.doi.org/10.1016/0376-6357(87)90035-0 (1987). 10.1016/0376-6357(87)90035-0 24925488

[b44] Begall S., Malkemper E. P., Červený J., Němec P. & Burda H. Magnetic alignment in mammals and other animals. Mamm. Biol. **78**, 10–20, http://dx.doi.org/10.1016/j.mambio.2012.05.005 (2013).

[b45] Begall S., Červený J., Neef J., Vojtěch O. & Burda H. Magnetic alignment in grazing and resting cattle and deer. Proceedings of the National Academy of Sciences 105, 13451–13455 (2008). 10.1073/pnas.0803650105 PMC2533210 18725629

[b46] Hart V. *et al.* Dogs are sensitive to small variations of the Earth's magnetic field. Front. Zool. 10, 80 (2013). 24370002 10.1186/1742-9994-10-80 PMC3882779

[b47] Ritz T. *et al.* Magnetic compass of birds is based on a molecule with optimal directional sensitivity. Biophys. J. 96, 3451–3457 (2009). 19383488 10.1016/j.bpj.2008.11.072 PMC2718301

[b48] Schulten K., Swenberg C. & Weller A. A biomagnetic sensory mechanism based on the geminate recombination of radical ion pairs in solvents. J. Phys. Chem. NF101, 371–390 (1978).

[b49] Xu B.-M., Zou J., Li H., Li J.-G. & Shao B. Effect of radio frequency fields on the radical pair magnetoreception model. Phys. Rev. E. 90, 042711 (2014). 10.1103/PhysRevE.90.042711 25375527

[b50] Lambert N., De Liberato S., Emary C. & Nori F. Radical-pair model of magnetoreception with spin-orbit coupling. New J. Phys. 15, 083024 (2013).

[b51] Nießner C. *et al.* Avian ultraviolet/violet cones identified as probable magnetoreceptors. PLoS. ONE 6, e20091 (2011). 21647441 10.1371/journal.pone.0020091 PMC3102070

[b52] Nießner C. *et al.* Magnetoreception: activated cryptochrome 1a concurs with magnetic orientation in birds. J. R. Soc. Interface 10, 20130638, 10.1098/rsif.2013.0638 (2013). 23966619 PMC3785833

[b53] Solov'yov I. A. & Schulten K. Magnetoreception through cryptochrome may involve superoxide. Biophys. J. 96, 4804–4813 (2009). 19527640 10.1016/j.bpj.2009.03.048 PMC2712043

[b54] Lee A. A. *et al.* Alternative radical pairs for cryptochrome-based magnetoreception. J. R. Soc. Interface **11**, 10.1098/rsif.2013.1063 (2014). PMC4006233 24671932

[b55] Wiltschko R., Stapput K., Thalau P. & Wiltschko W. Directional orientation of birds by the magnetic field under different light conditions. J. R. Soc. Interface 7, S163–S177 (2010). 19864263 10.1098/rsif.2009.0367.focus PMC2843996

[b56] Landler L., Painter M. S., Youmans P. W., Hopkins W. A. & Phillips J. B. Spontaneous magnetic alignment by yearling snapping turtles: rapid association of radio frequency dependent pattern of magnetic input with novel surroundings. PLoS. ONE. in press (2015). 10.1371/journal.pone.0124728 PMC4433231 25978736

[b57] Gauger E. M., Rieper E., Morton J. J. L., Benjamin S. C. & Vedral V. Sustained quantum coherence and entanglement in the avian compass. Phys. Rev. Lett. 106, 040503 (2011). 21405313 10.1103/PhysRevLett.106.040503

[b58] Phillips J. B., Muheim R. & Jorge P. E. A behavioral perspective on the biophysics of the light-dependent magnetic compass: a link between directional and spatial perception? J. Exp. Biol. 213, 3247–3255 (2010). 20833916 10.1242/jeb.020792

[b59] Červený J., Begall S., Koubek P., Nováková P. & Burda H. Directional preference may enhance hunting accuracy in foraging foxes. Biol. Lett. 7, 355–357, 10.1098/rsbl.2010.1145 (2011). 21227977 PMC3097881

[b60] Hart V. *et al.* Directional compass preference for landing in water birds. Front. Zool. 10, 38 (2013). 23835450 10.1186/1742-9994-10-38 PMC3710278

[b61] Kirschvink J. L. *et al.* in *Sensory Transduction*. (eds Corey D. P., & Roper S. D., eds. ) 225–240. (Society of General Physiologists, 45th Annual Symposium, Rockefeller University Press, 1992).

[b62] Fleissner G., Stahl B., Thalau P. & Falkenberg G. A novel concept of Fe-mineral-based magnetoreception: histological and physicochemical data from the upper beak of homing pigeons. Naturwissenschaften 94, 631–642 (2007). 17361399 10.1007/s00114-007-0236-0

[b63] ICNIRP. . in *Health Phys* Vol. 74 (ed International Commission for Non-Ionizing Radiation Protection.) 494–522 (1998). 9525427

[b64] Kirschvink J. L. Uniform magnetic fields and double wrapped coil systems: Improved techniques for the design of bioelectromagnetic experiments. Bioelectromagnetics 13, 401–411 (1992). 1445421 10.1002/bem.2250130507

[b65] Batschelet E. Circular Statistics in Biology. (Academic Press., 1981).

