# Supplementary material for: Magnetoreception in the wood mouse (Apodemus sylvaticus): influence of weak frequency-modulated radio frequency fields
Source: Sci Rep. 2015 Apr 29;4:9917. doi: 10.1038/srep09917 (PMC4413948; doi:10.1038/srep09917)
Supplement: Supplementary Information — Supplementary Table S1 [file srep09917-s1.doc]

**Magnetoreception in the wood mouse (*Apodemus sylvaticus*): influence of weak frequency-modulated radio frequency fields**

E. Pascal Malkemper, Stephan H. K. Eder, Sabine Begall, John B. Phillips, Michael Winklhofer, Vlastimil Hart, Hynek Burda

**Supplementary Table 1. Directions of single wood mice nests build in different magnetic conditions. LF = Larmor frequency, FM = frequency-modulated**

| Ambient field | West field | Ambient +  LF | Ambient +  Wideband-FM RF | |  |
| --- | --- | --- | --- | --- | --- |
| 0 | 55 | 45 | 135 |  | |
| 315 | 150 | 330 | 250 |  | |
| 185 | 155 | 315 | 160 |  | |
| 90 | 270 | 260 | 345 |  | |
| 35 | 335 | 25 | 120 |  | |
| 70 | 105 | 10 | 140 |  | |
| 90 | 5 | 245 | 20 |  | |
| 175 | 75 | 205 | 285 |  | |
| 65 | 120 | 145 | 120 |  | |
| 40 | 100 | 205 | 115 |  | |
| 35 | 195 | 160 | 90 |  | |
| 225 | 75 | 210 | 305 |  | |
| 185 | 110 | 195 | 205 |  | |
| 190 | 65 | 125 | 110 |  | |
| 225 | 305 | 165 | 150 |  | |
| 245 | 330 | 0 | 85 |  | |
| 355 | 250 | 175 | 150 |  | |
| 205 | 75 | 15 |  |  | |
| 320 | 345 | 20 |  |  | |
| 195 | 250 | 55 |  |  | |
| 255 | 310 | 195 |  |  | |
| 195 |  | 145 |  |  | |
| 180 |  |  |  |  | |
| 20 |  |  |  |  | |
|  |  |  |  |  | |
